# Prostatic Involvement in Granulomatosis with Polyangiitis: A Case Report and Scoping Review

**DOI:** 10.3390/diagnostics16193101

**Published:** 2026-09-24

**Authors:** Jakub Kulski, Kevin Miszewski, Oliwia Kałwak, Laura Miszewska, Bartłomiej Marczak, Michał Szczypior, Marcin Matuszewski

**Affiliations:** 1 Faculty of Medicine, Medical University of Gdańsk, 17 Smoluchowskiego St., 80-214 Gdańsk, Poland; jakub.kulski@gumed.edu.pl (J.K.);; 2 Department of Urology, Medical University of Gdańsk, 17 Smoluchowskiego St., 80-214 Gdańsk, Poland; 3 Faculty of Medicine, Medical University of Warsaw, 61 Zwirki i Wigury St., 02-091 Warsaw, Poland

**Keywords:** granulomatosis with polyangiitis, prostate, urogenital, ANCA-associated vasculitis, case report, scoping review, diagnostic delay

## Abstract

**Background and Clinical Significance:** Granulomatosis with polyangiitis (GPA) is a systemic antineutrophil cytoplasmic antibody (ANCA)-associated vasculitis that typically affects the respiratory tract and kidneys. Prostatic involvement is rare and may mimic infection or malignancy, delaying diagnosis. We report a case of exceptionally extensive urogenital destruction and provide a scoping review of prostatic involvement in GPA published since the last comprehensive review in 2019. **Case Presentation:** A 60-year-old man underwent repeated antibiotic courses and urological procedures for sterile, antibiotic-refractory prostatic and periurethral necrosis, initially attributed to infection or malignancy. Imaging showed a contiguous necrotising process extending from the prostatic fossa to the urethra, bladder trigone, penile base, and left upper urinary tract. With concurrent sinonasal and mastoid disease, karyorrhectic necrosis on histology, and low-titre ANCA positivity, GPA was diagnosed and treated with glucocorticoids and cyclophosphamide followed by rituximab, with regression of active disease but persistent structural sequelae. A PRISMA-ScR–guided search of PubMed, Scopus, Web of Science and Embase (24 August 2026) identified post-2019 reports of prostatic involvement in GPA. Fifteen publications plus the present case yielded 16 cases for structured extraction. Mean age at first prostatic manifestation was 55.1 years, and the mean reported interval to diagnosis was 4 months. Prostatic abscess was the most frequently reported imaging finding. PR3-ANCA positivity (13/16) and pulmonary involvement (13/16) were reported most often; renal involvement was reported in 4/16 cases and death in 4/16. Rituximab was reported more often than cyclophosphamide (10/16 vs. 7/16). **Conclusions:** Reports of prostatic involvement published from 2019 onward appear broadly consistent with the profile described in the last comprehensive review. These proportions, however, describe a small, publication-selected case series and are not estimates of the characteristics, prognosis or treatment response of prostatic involvement in GPA. Prostatic involvement may extend contiguously into the urethra, bladder, penile structures and upper urinary tract; the present case is one of the most extensive examples identified in this review. Sterile, antibiotic-refractory urogenital lesions, especially with extra-urogenital disease, low-titre or discordant ANCA serology, should prompt consideration of GPA before irreversible anatomical loss.

## 1. Introduction

Granulomatosis with polyangiitis is a rare systemic autoimmune vasculitis associated with anti-neutrophil cytoplasmic antibodies (ANCA) [1]. GPA classically manifests as a triad of respiratory tract granulomatous inflammation, systemic small-vessel vasculitis, and necrotising glomerulonephritis [2]. As a multisystemic disorder, GPA can involve virtually any organ, producing a highly heterogeneous clinical spectrum that extends well beyond the canonical triad [3]. Among its atypical manifestations, urogenital involvement is exceptionally rare, estimated to occur in fewer than 1% of patients [4,5,6,7]. Within the urogenital tract, prostatic involvement (PI) is the most frequently reported urological manifestation, although other reported presentations include orchitis, epididymitis, renal pseudotumours, ureteral stenosis, and cystitis [8]. Critically, urogenital involvement may precede the classic manifestations of GPA, so that in the absence of the characteristic triad it often mimics infection or urogenital malignancy and poses a diagnostic challenge [7,9,10]. If left untreated, progressive granulomatous inflammation and necrotising vasculitis may cause devastating and irreversible anatomical destruction [2,11]. On the other hand, when correctly identified, urogenital lesions are generally responsive to standard GPA remission-induction protocols combining glucocorticoids with cyclophosphamide or rituximab [2,9].

We present the case of a 60-year-old male with prostatic involvement in GPA complicated by an exceptional degree of contiguous urogenital destruction, and provide an updated synthesis of cases reported since the last comprehensive review of PI in GPA, published by Yatsyshyn et al. in 2019 [9]. The objectives of the review component were to identify and chart all case-level reports of PI in GPA published from 1 January 2019 onward, to describe their clinical presentation, diagnostic pathway, ANCA profile, treatment and reported outcomes, and to compare these descriptively with the earlier synthesis. A scoping-review design was chosen because the evidence consists exclusively of case reports and small series, which precludes formal effect estimation and critical appraisal of the kind undertaken in a systematic review.

## 2. Case Presentation

A 60-year-old male was admitted in February 2025 to the Department of Otolaryngology at the University Clinical Center in Gdańsk, presenting with progressive hearing loss in the right ear and an escalating, painful right-sided postauricular oedema, both persisting since December 2024. A chronological summary of the entire clinical course is provided in Table 1. The patient’s medical history was notable for a 10 kg weight loss, a diagnosis of sarcoidosis in 2011 (reported by the patient; no documentation was available and no treatment or recurrence had occurred since), and frequent travel to tropical regions. In November 2024, he underwent left-sided ureterorenoscopy (URS-L) at an external facility for hydronephrosis and suspected ureteric calculus. During the procedure, necrotic tissue was identified within the prostatic urethra. Postoperatively, the patient developed acute urinary retention (AUR), necessitating the placement of a suprapubic cystostomy. This catheter was subsequently removed in January 2025 following a transurethral resection of the prostate (TURP) performed to drain a suspected prostatic abscess. Histopathological examination revealed advanced granulomatous inflammation characterised by extensive karyorrhectic necrosis. Despite intensive antibiotic therapy (levofloxacin, metronidazole), the urological symptoms failed to resolve. Upon admission, laboratory investigations demonstrated a C-reactive protein (CRP) of 96.3 mg/L. Haematological findings included a haemoglobin level of 11.3 g/dL, reactive thrombocytosis at 805 × 10^9^/L, and peripheral eosinophilia (1.14 × 10^9^/L). Computed tomography (CT) of the temporal bones revealed a subperiosteal abscess (33 mm × 16 mm × 30 mm) of the right mastoid process (Figure 1). Under local anaesthesia, paracentesis of the tympanic membrane was performed, followed by aspiration of the postauricular abscess. Specimens were submitted for microbiological analysis. Empirical antibiotic therapy with ceftriaxone was initiated, and in February a right-sided antromastoidectomy was performed. Cultures from both the ear and the abscess were negative for bacterial growth. Microscopic evaluation demonstrated areas of karyorrhectic necrosis and distorted fibrous tissue within an inflammatory exudate characterised by marked eosinophilic infiltration. On the first postoperative day, the patient reported perineal pain and discomfort, purulent urethral discharge, and progressive impairment of bladder emptying. Consequently, a concurrent urological diagnostic workup was initiated. Urinalysis revealed features of active inflammation (leukocyturia +++, erythrocyturia ++, proteinuria +, nitrites negative). Expanded diagnostic testing for infectious aetiologies, including urine cultures, serological and molecular testing for atypical infections (*Mycoplasma pneumoniae*, *Ureaplasma urealyticum*, *Chlamydia trachomatis*) and tuberculosis, yielded negative results. CT imaging identified an abscess within the prostatic fossa measuring 5.8 cm × 5.2 cm × 6.5 cm (Figure 2). Additionally, thickening of the posterior bladder wall was noted, suggestive of an inflammatory or neoplastic process, alongside left-sided nephrolithiasis and ureterolithiasis with associated hydronephrosis. The patient was subsequently scheduled for transurethral drainage of the abscess (March 2025). Intraoperatively, massive fibrin deposits and necrotic material were observed extending from the bulbar urethra to the bladder, which precluded identification of the true urethral lumen (Figure 3). Because it was impossible to access the urinary bladder endoscopically, a suprapubic cystostomy had to be reinserted to secure urinary diversion. Microscopic examination once again demonstrated necrotic-karyorrhectic material and an inflammatory infiltrate characterised by significant eosinophilic infiltration; atypia and malignancy were notably absent. Despite broad-spectrum antibiotic therapy, after three weeks, the patient experienced a recurrence of the painful right-sided postauricular oedema. Incision and drainage of the lesion were performed. Persistently high inflammatory markers (CRP up to 183 mg/L) were observed, alongside leucocytosis, eosinophilia (~1.1 ×10^9^/L), normocytic anaemia, and thrombocytosis. High-resolution CT (HRCT) of the temporal bones demonstrated progressive destruction of the right mastoid air cell trabeculae. Contrast-enhanced maxillofacial CT revealed nodular mucosal thickening of the nasal septum and the right maxillary sinus ostium characterised by intense post-contrast enhancement (Figure 4). A chest CT demonstrated calcified hilar and mediastinal lymphadenopathy without parenchymal abnormality; the nodes were unchanged in size compared with the 2011 scan. Simultaneously, the patient’s perineal pain and purulent urethral discharge intensified. MRI of the abdomen and pelvis visualized an extensive, polycyclic inflammatory-necrotic collection in the lesser pelvis (maximum dimensions 12 cm × 6.5 cm × 5.5 cm) originating from the prostate and involving the bulb and base of the penis, the bladder trigone, and the periurethral region (Figure 5). Circumferential infiltration of the left ureteropelvic junction with secondary hydronephrosis was also identified. Given its radiological resemblance to a proliferative urothelial neoplasm, direct endoscopic evaluation was mandated. A left-sided percutaneous nephrostomy (mini-PCN) was established, allowing the introduction of a nephroscope to directly visualize an exophytic lesion at the ureteropelvic junction. Targeted biopsies were obtained, and a 14Fr nephrostomy tube was left in place to secure urinary drainage. Crucially, histopathological verification definitively ruled out malignancy, revealing instead fibrous tissue with suppurative-necrotic inflammatory infiltration, a morphological picture mirroring the necrotic destruction previously observed within the prostate. Following the failure of the prior transurethral drainage attempt, open perineal drainage of the pelvic and perineal abscess was performed in April 2025. The procedure included resection of the necrotic-infiltrative tissues involving the urethra and the prostatic region (Figure 6). Multidisciplinary consultations (urology, otolaryngology, rheumatology, immunology, haematology, microbiology and pulmonology) were subsequently sought. Differential diagnosis effectively ruled out haematological malignancies (negative *JAK2*, *CALR*, and *MPL* mutations; absence of monoclonal protein on immunofixation), primary immunodeficiencies (normal immunoglobulin levels, lymphocyte subpopulations, and dihydrorhodamine (DHR) test), and IgG4-related disease. Testing for tropical pathogens and tuberculosis (QuantiFERON) also remained negative. A pulmonology consultation on 7 April 2025 found no clinical or radiological evidence of active pulmonary sarcoidosis. ANCA testing was performed on 8 April 2025. Indirect immunofluorescence (IIF; EUROIMMUN granulocyte substrate mosaic, Lübeck, Germany) showed a cytoplasmic pattern (c-ANCA) at a titre of 1:40 (negative < 1:40), whereas quantitative PR3-ANCA by fluorescence enzyme immunoassay (EliA PR3S, Phadia 250, Thermo Fisher Scientific, Uppsala, Sweden) was 1.80 IU/mL, within the manufacturer’s negative range (<2.0 IU/mL), and MPO-ANCA was 0.20 IU/mL (<3.5 IU/mL). A second, antigen-specific assay was performed on 22 April 2025 (ANCA-Profile ELISA (IgG), EUROIMMUN; six separately coated antigens, PR3 as native and recombinant human protein; results as the ratio to cut-off): the PR3 ratio was 2.50 (positive 2.0–5.0), with negative results for MPO, lactoferrin, elastase, cathepsin G and BPI. At a joint rheumatology–immunology consultation on 28 April 2025, with neoplastic and infectious causes excluded, ANCA-associated vasculitis was suspected on the basis of the clinical picture (recurrent, sterile, antibiotic-refractory necrotising lesions of the mastoid and pelvis with sinonasal disease on imaging, and concordant histology of karyorrhectic necrosis with inflammatory infiltrate) together with the presence of ANCA at low titre. Systemic immunosuppressive therapy was initiated on the same day with oral prednisone (60 mg/day), resulting in rapid clinical and laboratory improvement (CRP 19 mg/L). An attempt to administer intravenous immunoglobulin (IVIG, 2 g/kg) was discontinued because of adverse effects. On 5 May 2025, one week into glucocorticoid therapy, the IIF titre had risen to 1:80 while PR3-ANCA remained below threshold (1.40 IU/mL). After four weeks of prednisone, the ANCA-profile ELISA had become negative (PR3 ratio 0.50 on 27 May 2025). On 28 May 2025, the patient was admitted to the Department of Rheumatology to establish the definitive diagnosis and modify treatment. On the basis of the entire clinical picture, systemic ANCA-positive GPA was diagnosed. The serological findings, a c-ANCA pattern on IIF with PR3 reactivity on the second immunoassay but a primary quantitative assay below its threshold, were regarded as low-titre ANCA positivity supporting, rather than establishing, the diagnosis. Remission induction with intravenous cyclophosphamide according to the EUVAS pulse regimen was started (first pulse 1000 mg, i.e., 12.5 mg/kg, reduced for leukopenia; six pulses to September 2025; cumulative dose 7.2 g), with mesna, intravenous hydration, trimethoprim/sulfamethoxazole prophylaxis and a tapering glucocorticoid course. Within several weeks of treatment, significant clinical improvement was achieved, including regression of otolaryngological lesions and marked reduction in urological symptoms. A decline in inflammatory markers, normalization of eosinophil counts, and gradual improvement in haematological parameters were noted. At the time of the sixth pulse (September 2025), ANCA were undetectable by IIF (<1:40) and PR3-ANCA was 0.20 IU/mL. Because of the granulomatous mastoid and pelvic disease, maintenance rituximab was subsequently introduced (1000 mg in November and December 2025, and again in May and June 2026). Follow-up pelvic MRI (March 2026) and CT (April 2026) showed complete radiological regression of the prostatic-fossa collection, with no residual abscess. Regression of active disease, however, must be distinguished from the structural sequelae of the pre-treatment destruction. Two weeks after removal of the urethral catheter (June 2025), the patient developed urinary retention that could not be relieved by catheterisation, and a suprapubic cystostomy was re-established. Left-sided orchitis followed in June 2025. Voiding cystourethrography (9 July 2025) showed a post-abscess cavity with an intracavitary polypoid mass and a 9 mm-long bulbar urethral stricture of approximately 2 mm calibre. A Th7 vertebral compression fracture identified in August 2025 was attributed to remote trauma rather than glucocorticoid-induced osteoporosis. An obstructing left ureteropelvic-junction calculus required percutaneous nephrostomy (September 2025) and nephrolithotripsy with ureteral stenting (November 2025). An extended-spectrum β-lactamase-producing *Pseudomonas aeruginosa* urinary tract infection (October 2025; CRP 154 mg/L with negative ANCA) required inpatient intravenous antibiotics, and a retroauricular fistula was closed surgically in November 2025. These complications were managed conservatively and surgically without interrupting immunosuppression. At the most recent follow-up (June 2026), the patient was in clinical remission (BVAS/WG 0) with no new organ involvement. He remained dependent on suprapubic urinary diversion.

## 3. Materials and Methods

### 3.1. Study Design

The case is reported in accordance with the CARE guidelines for case reports [12], including a structured clinical timeline (Table 1). The literature component was designed as a scoping review of case-level evidence and is reported according to the Preferred Reporting Items for Systematic Reviews and Meta-Analyses extension for Scoping Reviews (PRISMA-ScR) [13]; the completed checklist is provided as Supplementary Table S1. No review protocol was registered. The review was conceived as an update of the systematic case collation published by Yatsyshyn et al. in 2019, who searched MEDLINE/PubMed, Scopus, Google Scholar and HINARI and analysed 45 published cases of PI in GPA [9]. The eligibility period of the present review therefore begins on 1 January 2019, the calendar year of that publication, so that the two syntheses are contiguous. In the event, all 15 included reports were published in 2020 or later, and none had been included by Yatsyshyn et al. The consequences of this design choice are considered in the Limitations.

### 3.2. Literature Search

PubMed and Scopus were searched on 30 April 2026. In response to peer review, the same two-concept search was executed in Web of Science Core Collection and Embase (Elsevier) on 24 August 2026, and the PubMed and Scopus strategies were re-run on the same date. Neither re-run retrieved any record not already retrieved by the original strategy (Supplementary Material S1). No language restrictions were applied. The publication window was limited to 1 January 2019 onward. The search strategy combined two concept blocks with Boolean operators: the disease block (“granulomatosis with polyangiitis”, the historical eponym “Wegener’s granulomatosis”/”Wegener granulomatosis”, and “ANCA-associated vasculitis”) and the anatomical block (“prostate”, “prostatic”, “prostatitis”). Search fields were Title/Abstract in PubMed, Title/Abstract/Keywords (TITLE-ABS-KEY) in Scopus, Topic (title, abstract, author keywords, Keywords Plus) in Web of Science, and title/abstract/keyword fields plus exploded Emtree headings in Embase, where conference abstracts were excluded at the search stage. This restriction was chosen to maximise specificity for reports in which PI was a substantive finding rather than an incidental mention. The full electronic search strategies, exactly as executed, with the number of records retrieved per database, are reproduced in Supplementary Material S1.

### 3.3. Eligibility Criteria

Eligible sources included case reports, case series, clinical vignettes, brief communications, and letters to the editor containing original patient data, provided that sufficient clinical, laboratory, and histopathological information was available to allow extraction of relevant variables. Conference abstracts and publications lacking extractable data were excluded. A diagnosis of GPA was accepted as established by the authors of the source report when it rested on a combination of compatible clinical features, ANCA serology and/or histopathology, with GPA stated as the final diagnosis of the treating clinicians.

### 3.4. Selection Process

Records were exported from each database and deduplicated in Zotero by automated matching of DOI, title and first author, followed by manual verification of all flagged pairs. All records identified through database searches were screened by two independent reviewers (J.K., K.M.). Titles and abstracts were initially assessed, followed by full-text evaluation of potentially eligible reports. Disagreements were resolved by discussion. No third-party arbitration was required. The numbers of records identified, screened, assessed and included are reported in Section 4.1 and in the PRISMA-ScR flow diagram.

### 3.5. Data Extraction

Data extraction was performed using a predefined data collection form. Data were charted by one reviewer and independently verified by a second (J.K., K.M.); discrepancies were resolved by discussion. Extracted variables included patient age, time to diagnosis, presenting urological symptoms, imaging findings regarding prostate involvement, extra-prostatic organ involvement, ANCA profile, immunosuppressive treatment, surgical interventions, and clinical outcomes.

The following operational definitions were applied. *Age* was recorded at the first prostatic manifestation. *Time to diagnosis* was defined as the interval from the first documented urological symptom or prostatic abnormality attributable to PI to the date on which GPA was diagnosed, as directly calculable from the clinical course described by the authors. Where the date of diagnosis could not be established, the date of initiation of GPA-directed treatment was used instead. Intervals given as “<1 month” or “two weeks” were coded as 0.5 months, and cases in which none of these dates could be determined were recorded as “not reported” and excluded from the summary statistic. *Urological symptoms* were classified as presenting when they constituted the initial complaint or arose concurrently with the systemic presentation. *Organ involvement* was recorded as attributed to GPA by the reporting authors. *Renal involvement* required necrotising glomerulonephritis or interstitial nephritis, confirmed by biopsy or autopsy and attributed to GPA; acute kidney injury without histopathological confirmation was recorded separately and excluded from the renal subgroup. *ANCA profile* was recorded as reported; results not reported were coded as not available. *Outcome* was recorded at the last reported follow-up as death; remission, when the authors explicitly reported clinical and/or radiological remission with a stated follow-up duration or improvement, when clinical, laboratory or radiological improvement was reported without an explicit statement of remission or with a follow-up too short to establish it. Non-English articles were translated to enable extraction of relevant clinical data: one French-language article by a French-speaking author, and one Russian-language article using two large language models independently (listed in the Acknowledgements). The two machine translations were compared sentence by sentence by one reviewer (J.K.); numerical data (age, laboratory values, doses, dates), which are language-independent, were verified directly against the original text, and all discrepancies concerned wording only and none affected any charted variable. Charted data were synthesised descriptively as frequencies and proportions (n/N, %) and, for age and time to diagnosis, as mean, median and range. No inferential statistics, meta-analysis or quantitative pooling beyond this descriptive case-level synthesis was undertaken, and, consistent with scoping-review methodology, no critical appraisal of individual sources was performed [13]. All extracted data are summarized in Table 2.

## 4. Results

### 4.1. Selection of Sources of Evidence

A total of 234 records were identified: 22 in PubMed, 91 in Scopus, 25 in Web of Science and 96 in Embase. After removal of 124 duplicates, 110 records were screened by title and abstract, of which 90 were excluded. The remaining 20 reports were assessed in full text; five were excluded (four for insufficient urological data, one because the PI was not confirmed), leaving 15 publications [14,15,16,17,18,19,20,21,22,23,24,25,26,27,28] in the final analysis (Figure 7). The searches of Web of Science and Embase and the re-executed PubMed and Scopus strategies, added during peer review, identified one additional report for full-text assessment, which was excluded for insufficient urological data; no additional eligible report was found. Combined with the present case, the charted series comprised 16 cases, each contributed by a different publication.

### 4.2. Demographics and Time to Diagnosis

Mean age at first prostatic manifestation was 55.1 years (median 58, range 26–78). The interval between symptom onset and formal GPA diagnosis was reported or calculable in 13/16 (81.3%) cases. In these, the mean time to diagnosis was 4 months (median 3, range 0.5–9). In the present case the interval was seven months, from the first documented prostatic abnormality (November 2024) to the formal diagnosis (May 2025). Urological symptoms preceded or co-dominated the presenting illness in 12/16 (75%) cases.

### 4.3. Urological Symptoms and Structural Findings

The urological symptom profile was heterogeneous. Dysuria was the most frequently reported complaint (8/16, 50.0%), followed by haematuria (6/16, 37.5%), acute urinary retention (5/16, 31.3%), and pollakiuria (5/16, 31.3%). Urethral discharge or bleeding was reported in 3/16 (18.8%), bladder outlet obstruction in 3/16 (18.8%), and pelvic pain in 2/16 (12.5%). Two patients (2/16, 12.5%) were entirely asymptomatic from a urological standpoint, with prostatic involvement detected during evaluation of pulmonary or systemic disease. Prostatic imaging was obtained in 13/16 (81.3%) cases. The most frequently reported structural finding was prostatic abscess (8/13, 61.5%), followed by prostatic enlargement (5/13, 38.5%) with concurrent abscess and enlargement in 2 cases [18,27]; two further cases showed neither: prostatic calcifications [16] and FDG-avid nodular foci [22]. Extension of the granulomatous-necrotising process beyond the prostate was documented in a subset of reports. Bladder involvement was present in 4/16 (25.0%) cases, including the present case [19,26,27]. Direct urethral involvement, ranging from periurethral inflammation to frank urethral necrosis, was present in 3/16 (18.8%) cases, also including the present case [23,28]; one additional patient [20] developed a catheterisation-related urethral stricture and was excluded from this count. Among the 16 charted cases, the present case is distinguished by the anatomical extent of contiguous urological destruction, encompassing prostatic abscess, urethral necrosis, bladder trigone infiltration, penile involvement, and circumferential infiltration of the left ureteropelvic junction with secondary hydronephrosis. To our knowledge, no previously reported case has documented this combination of contiguous urogenital involvement, although the search strategy does not permit this to be stated with certainty (see Section 6).

### 4.4. Extra-Prostatic Organ Involvement

Pulmonary involvement was the most frequently reported manifestation (13/16, 81.3%), followed by constitutional or systemic features (11/16, 68.8%) and ENT involvement (9/16, 56.3%). Renal involvement, as defined in Section 3.5, was reported in 4/16 (25.0%) cases; all four met the definition through histological confirmation, although the patterns differed. Pauci-immune necrotising glomerulonephritis was confirmed by biopsy in one case [24], while autopsy demonstrated necrotising glomerulonephritis in two further cases, crescentic in one [26] and showing focal fibrinoid glomerular necrosis in the other [27]. The remaining case demonstrated acute interstitial nephritis without glomerulonephritis [25]. One additional patient with acute kidney injury and no histopathological confirmation did not meet this definition and was not counted as renal involvement [18]; the case is labelled “AKI without histological confirmation” in Table 2. Ocular involvement, including episcleritis, panuveitis, and conjunctival hyperaemia, was documented in 4/16 (25%) cases [14,16,21,26]. CNS involvement was documented in 3/16 (18.8%) cases with two distinct patterns: granulomatous (hypophysitis with central diabetes insipidus [16]; intracranial granuloma during rituximab maintenance [24]) and vasculitic-thromboembolic (multiple cerebral infarctions progressing to fatal subcortical haemorrhage [26]). Articular manifestations were noted in 2/16 (12.5%) cases [14,18], cutaneous involvement in 2/16 (12.5%) [17,20], and splenic involvement in 2/16 (12.5%) [22,26].

### 4.5. Immunological Profile

ANCA serology was reported with variable completeness across cases. By indirect immunofluorescence (IIF), c-ANCA positivity was documented in 11/16 (68.8%) cases, while p-ANCA positivity was reported in one case (1/16, 6.3%). By antigen-specific immunoassay, PR3-ANCA was reported as positive in 13/16 (81.3%) cases, whereas MPO-ANCA positivity was identified in only one patient (1/16, 6.3%), representing the only reported p-ANCA/MPO-ANCA-positive profile in this series [21]. Assay cut-offs were reported in a few of the published cases, so ANCA positivity in Table 2 reflects the designation of the reporting authors.

### 4.6. Therapeutic Interventions

Glucocorticoids were administered in all 16 cases (100%). Glucocorticoid monotherapy was used in 2/16 (12.5%) cases [26,27]; both patients died in the acute phase before adjunctive immunosuppression could be initiated. Rituximab was the most frequently reported steroid-sparing agent (10/16, 62.5%), followed by cyclophosphamide (7/16, 43.8%). The rituximab count includes use for induction, maintenance and relapse, which the source reports did not always distinguish. Three patients received both agents sequentially, cyclophosphamide for induction, rituximab for maintenance or at relapse [17,24], including the present case. Azathioprine featured in 4/16 (25.0%) cases as maintenance or relapse therapy, methotrexate in 2/16 (12.5%), mycophenolate mofetil in 1/16 (6.3%), and plasma exchange in 1/16 (6.3%). Surgical or procedural urological intervention was performed in 9/16 (56.3%) cases: transurethral resection of the prostate in 5/16 (31.3%), abscess drainage (transrectal, percutaneous, or open) in 5/16 (31.3%), and urinary diversion via suprapubic cystostomy or catheter in 4/16 (25.0%). The present case required the most extensive surgical management among the 16 cases: TURP, percutaneous nephrostomy, suprapubic cystostomy, and open perineal drainage with urethral resection.

### 4.7. Outcomes

Death was reported in 4/16 cases (25%): diffuse alveolar haemorrhage refractory to glucocorticoids, cyclophosphamide, and plasmapheresis [18]; fatal subcortical haemorrhage on day 13 of treatment [26]; cardiac arrest one day after diagnosis [27]; and an uncertain cause one week after rituximab induction in a patient with progressive pulmonary GPA [15]. All deaths occurred during or immediately after induction therapy. Two of the four fatal cases received glucocorticoid monotherapy at the time of death, while the remaining two died despite the initiation of definitive immunosuppression (cyclophosphamide or rituximab). Remission, as defined in Section 3.5, was reported in 5/16 cases (31.3%), including the present case, with reported follow-up ranging from 6 months to 5 years. Improvement without an explicit statement of remission was reported in 7/16 (43.8%) cases, in most of which follow-up was either not stated or too short to determine remission status. Because follow-up was heterogeneous and frequently brief, these figures describe what was reported at the time of publication and cannot be interpreted as mortality, remission or treatment-response rates. The present case achieved clinical remission with regression of the active ENT and pelvic inflammatory lesions, but the post-induction course was complicated by persistent structural sequelae (urethral stricture, dependence on suprapubic diversion), recurrent urinary retention, urinary tract infections, left-sided obstructive uropathy requiring nephrostomy, left orchitis, and a Th7 vertebral compression fracture (most likely of remote traumatic origin).

## 5. Discussion

Diagnostic delay in GPA is generally substantial, with reported median intervals from symptom onset to diagnosis of three to six months [29,30]; in a large multi-vasculitis cohort, initial misdiagnosis exceeded 70% [29]. The mean interval to diagnosis in the 13 cases with available data, 4 months in the charted cases, is therefore not exceptional in absolute terms. What appears to distinguish the urological presentation is the nature of medical activity that takes place during this interval. In pulmonary or renal GPA, the period preceding diagnosis is typically characterised by progressive symptomatology under observation, with typical diagnostic procedures like serology, imaging tests or biopsies contributing to recognition of the disease [2]. In the reports of PI charted here, by contrast, the same interval is occupied by a cascade of considerably more invasive interventions like TURP, abscess drainage, suprapubic cystostomy, ureteral stenting or partial penectomy [11], all performed under working diagnoses of infection or malignancy. In the case reported by Neerhut et al., rapidly recurrent prostatic obstruction necessitated two TURPs within four months before the diagnosis of GPA was established, with 44 g of granulomatous tissue resected in total [15]. In the case reported by Butt et al., repeated catheterisations performed for recurrent urinary retention produced a secondary urethral stricture [20]. Overall, surgical or procedural urological intervention was required in 9 of 16 cases. We interpret these interventions as carrying a structural cost that is independent of the underlying vasculitic process, and that may persist after immunological remission has been achieved, as in the present patient. This is an interpretation drawn from the case narratives rather than an established feature of urogenital involvement in GPA. No study has quantified procedural morbidity in this setting, and the present series cannot do so.

A second consequence of delayed recognition is that the disease, left without targeted immunosuppression, has time to extend. Granulomatous lesions in GPA may arise by contiguous extension from adjacent structures; most orbital involvement, for example, is considered to develop by direct spread from the paranasal sinuses [31] and in the review by Noda et al., the contiguous nature of granulomatous inflammation made urethral involvement difficult to separate clinically from adjacent penile or vaginal lesions [11]. The anatomical extent of urogenital involvement in the present case is consistent with this mechanism. A localized abscess restricted to the prostatic fossa expanded from 5.8 cm × 5.2 cm × 6.5 cm to a polycyclic collection reaching 12 cm × 6.5 cm × 5.5 cm over successive imaging studies; the urethra was completely obstructed by necrotic occlusion extending from the bulbar segment to the bladder neck; the bladder trigone and the left ureteric orifice were infiltrated; and the inflammatory process extended dorsally into the bulb and root of the penis. A synchronous but anatomically discontinuous stenosing lesion of the left ureteropelvic junction, radiologically indistinguishable from urothelial malignancy on diffusion-weighted imaging, produced obstructive uropathy requiring percutaneous nephrostomy. Among the cases of PI in GPA retrieved by our search, none documented this level of contiguous urogenital destruction extending across the prostate, urethra, bladder and penile structures within a single patient. The concurrent UPJ lesion, anatomically separate from the main process, suggests that the contiguous mechanism in this patient operated alongside multifocal disease activity rather than alone.

Deaths and long-term morbidity in the charted cases appear to follow different drivers, although the numbers are too small for this to be more than an observation. All four deaths occurred during or immediately after induction therapy and were systemic in mechanism (diffuse alveolar haemorrhage, intracranial haemorrhage, cardiac arrest, and progressive pulmonary disease); none was reported as primarily attributable to the prostatic lesion. Long-term morbidity, by contrast, where reported, was dominated by the local anatomical sequelae of pre-treatment destruction. On this reading, systemic disease determines whether the patient survives induction, the urogenital component determines the structural deficit the survivor is left with and the magnitude of that deficit is set during the diagnostic interval that precedes treatment.

Granulomatous prostatitis is not pathognomonic for GPA and requires systematic exclusion of mimics, including infectious and non-infectious diseases [32]. Infectious causes (tuberculous and atypical mycobacterial, fungal, schistosomal, brucellar, and syphilitic prostatitis) were excluded through negative cultures, repeated QuantiFERON testing, and targeted assays for atypical and tropical pathogens, the last prompted by the patient’s extensive travel history. Local non-infectious mimics, namely idiopathic and post-procedural granulomatous prostatitis, were rendered unlikely by the multisystem distribution of disease, the prostatic lesion having developed alongside sterile, antibiotic-refractory destructive mastoiditis and sinonasal disease.

Sarcoidosis warranted specific consideration: the patient carried a diagnosis from 2011, and sarcoidosis and ANCA-associated vasculitis appear as mutual alternative diagnoses in the work-up of granulomatous disease [33]. Active sarcoidosis was nonetheless excluded. The 2011 diagnosis was patient-reported and undocumented, had never required treatment and had not recurred; the only residuum was calcified hilar and mediastinal lymphadenopathy, unchanged in size since 2011, and a pulmonology consultation in April 2025 found no evidence of active pulmonary sarcoidosis. The histology was the opposite of what sarcoidosis requires. Its diagnosis rests on three criteria, a compatible presentation, non-necrotising granulomatous inflammation in one or more tissues and the exclusion of alternative causes [34], and sarcoid granulomas are structurally well formed and typically non-necrotising [33]. Extensive necrosis containing nuclear debris with a robust neutrophil- and eosinophil-rich inflammatory infiltrate, the pattern seen in all three specimens from this patient, is listed among the histological features that argue against sarcoidosis and for an alternative diagnosis [34]. ANCA, although at low level, were present. In the same differential, low titre ANCA positivity together with necrotising lesions points to ANCA-associated vasculitis rather than sarcoidosis [34].

Eosinophilic granulomatosis with polyangiitis (EGPA) was also considered, because peripheral eosinophilia (1.14 × 10^9^/L) and eosinophil-rich tissue infiltrates were present. EGPA was judged unlikely for several reasons. Asthma is present in more than 90% of patients with EGPA, ear, nose and throat disease (typically chronic rhinosinusitis with nasal polyposis) in 60–80% and peripheral neuropathy in 50–70% [35], all of which were absent in this patient. Eosinophilia exceeding 10% of leukocytes or 1.5 × 10^9^/L is observed in almost all patients with EGPA [35], whereas the eosinophilia here was modest (1.14 × 10^9^/L) and is documented in a substantial minority of patients with GPA in the French Vasculitis Study Group registry, where it identifies a subgroup with more severe disease [36]. ANCA are detectable in only 30–40% of patients with EGPA and are then almost always MPO-specific with a perinuclear pattern; PR3-ANCA-positive EGPA is rare and phenotypically closer to GPA [35], and c-ANCA/PR3 positivity is accordingly weighted against EGPA in the 2022 ACR/EULAR classification criteria [37].

Nevertheless, even once vasculitis was suspected, the serology did not straightforwardly confirm it. The pattern was one of low-level, discordant PR3-ANCA reactivity: c-ANCA was positive by IIF at 1:40, rising to 1:80 on repeat; the quantitative PR3-ANCA fluorescence enzyme immunoassay returned 1.80 and 1.40 IU/mL, below its 2.0 IU/mL cut-off; and a second, antigen-specific ELISA returned a PR3 ratio of 2.50, within its positive range, with the remaining antigens negative. All reactivity disappeared with treatment. The 2017 international consensus on ANCA testing recommends antigen-specific PR3- and MPO-ANCA immunoassays as the primary screening method and advises that, when clinical suspicion is high but the result is negative or low-positive, a second immunoassay and/or IIF be used, since a diagnosis of ANCA-associated vasculitis cannot be excluded on the basis of negative results [38]. The diagnostic pathway in our patient followed this logic, but the outcome should not be over-read: two of three methods indicated PR3 reactivity at a low level, the primary quantitative assay did not, and the diagnosis rested on the total clinical scenario, the concordant necrotising histopathology at two remote sites, the exclusion of mimics and the treatment response, with serology in a supporting role. This pattern has a precedent in the urethral GPA literature: Ahmed et al. observed that among 14 patients with urethral involvement in whom ANCA were measured, titres were ≤1:80 in all but two, and noted that urogenital manifestations may not be associated with high ANCA titres [39].

The demographic, serological and extra-prostatic profile of the 16 charted cases follows the general pattern of GPA rather than suggesting a distinct phenotype. The following comparisons with larger cohorts are indirect, and none implies that the 16 cases are representative of patients with PI, let alone of GPA in general. Mean age at first prostatic manifestation was 55.1 years, comparable both with the Yatsyshyn cohort (50.6 ± 12.4 years) [9] and with the typical age at diagnosis in unselected GPA populations [4,40]. PR3-ANCA positivity was reported in 13 of 16 patients (81.3%), with one MPO-positive case [21]. This PR3 predominance is in keeping with GPA, in which PR3-ANCA is found in approximately two-thirds to three-quarters of cases [1]. Pulmonary involvement was the most frequently reported manifestation (13/16, 81.3%), a proportion similar to that in the French Vasculitis Study Group registry (67.7% of 727 patients) [41]. ENT involvement was documented in 9 of 16 patients (56.3%), below the approximately 80% reported in that registry. The largest numerical difference concerned the kidneys: renal involvement was reported in 4 of 16 patients (25.0%), compared with 56.9% in the registry [40,41] and 40–100% across older cohorts [4,5,6], and close to the 22% reported by Yatsyshyn et al. [9]. The similarity between the two literature-based series is noted as a hypothesis-generating observation. It may reflect a genuine feature of PI, but it is equally consistent with reporting bias, since cases in which prostatic disease dominates the presentation are the ones most likely to be written up under this heading, whereas patients with prominent renal disease and incidental prostatic findings may be reported, if at all, under a different rubric. With 16 cases, the observation cannot be taken further.

Rituximab was reported more often than cyclophosphamide in the cases published from 2019 onward (10 of 16 versus 7 of 16), whereas the cases collated before 2019 were predominantly treated with cyclophosphamide [9]. This is a descriptive observation about a selected case series, not a treatment cohort, and the rituximab count includes maintenance and relapse therapy as well as induction. The difference is plausibly explained by contemporary practice and by reporting patterns rather than by any property of PI. Contemporary practice has itself changed. The RAVE and RITUXVAS trials established rituximab as non-inferior to cyclophosphamide for remission induction in severe ANCA-associated vasculitis [42,43], and this has been incorporated into subsequent guidance: the 2022 EULAR recommendations endorse glucocorticoids combined with either agent for organ-threatening GPA, with rituximab preferred in relapsing disease [44], and the 2021 ACR/Vasculitis Foundation guideline conditionally recommends rituximab over cyclophosphamide for remission induction in severe GPA [45]. This change is driven largely by the toxicity of cyclophosphamide, notably the dose-dependent risk of bladder malignancy and haemorrhagic cystitis with cumulative exposure, and gonadal toxicity [42,44].

Whether this preference extends to predominantly granulomatous, mass-forming disease such as urogenital involvement in GPA is less certain. Granulomatous manifestations are sometimes regarded as less responsive to B-cell depletion than vasculitic ones [46,47], but these signals derive from other granulomatous compartments rather than urogenital disease, and no study has compared rituximab with cyclophosphamide specifically in urogenital or prostatic involvement. The charted series is too small and too heterogeneous in follow-up to address the question, which would warrant dedicated comparative study.

## 6. Limitations

Several limitations apply to this analysis. First, the review was designed as an update of the synthesis by Yatsyshyn et al. [9] and therefore covers only reports published from 1 January 2019 onward. This design makes the completeness of the present series dependent on the completeness of that earlier review for the pre-2019 literature, and it means that the present review cannot independently characterise the entire published experience of PI in GPA. We accepted this trade-off because the aim of the present work was to describe what has been added since, not to replace that synthesis. Second, the search was originally confined to PubMed and Scopus and was extended to Web of Science and Embase, with controlled-vocabulary and spelling variants, during peer review. This extension retrieved no additional eligible report, which is reassuring but does not exclude omissions, because the search was restricted to title, abstract and keyword fields; reports mentioning PI only in the body of the text, or indexed under different subject headings, may therefore have been missed. Third, the included cases are drawn from a literature dominated by single-patient reports, with heterogeneous reporting standards for clinical, serological and follow-up data. The proportions reported here (for example, PR3-ANCA positivity, pulmonary or renal involvement, death or remission) describe what was reported in these 16 cases and are not estimates of the frequency, prognosis or treatment response of PI in GPA. Selection and publication bias toward more severe, more unusual or more instructive cases is likely, and the absence of any registry-based estimate of prostatic involvement in GPA leaves the true population frequency unknown. Comparison with the Yatsyshyn series and with registry cohorts is therefore descriptive and indirect rather than statistical. Follow-up duration varies widely and is often insufficient to determine long-term remission status or late sequelae. Fourth, one Russian-language report was translated with the aid of large language models rather than by a professional translator, as described in Section 3.5. Finally, in the present case the serological evidence for GPA was low-level and discordant across assays, and the diagnosis rests on the aggregate clinical, histopathological and therapeutic picture rather than on conclusive serological proof.

## 7. Conclusions

The cases of PI in GPA published from 2019 onward, together with the present case, appear broadly consistent with the pattern described by Yatsyshyn et al. Two further observations emerge from the charted reports. First, PI can extend contiguously into the urethra, bladder, penile structures and upper urinary tract. The present case is one of the most extensive cases identified in the present review, and we regard contiguous spread as a possible pattern of extension that merits attention in future reports. Second, rituximab was reported more often than cyclophosphamide in these recent reports, in line with contemporary guidance, although the series is too small and too selected to support conclusions about comparative efficacy in urogenital disease. Across both features, the recurring lesson is diagnostic: sterile, antibiotic-refractory granulomatous-necrotic urogenital lesions, particularly when accompanied by extra-urogenital disease, low-titre or discordant ANCA serology, should prompt consideration of GPA before the diagnostic interval translates into irreversible anatomical loss.

## Figures and Tables

**Figure 1:**
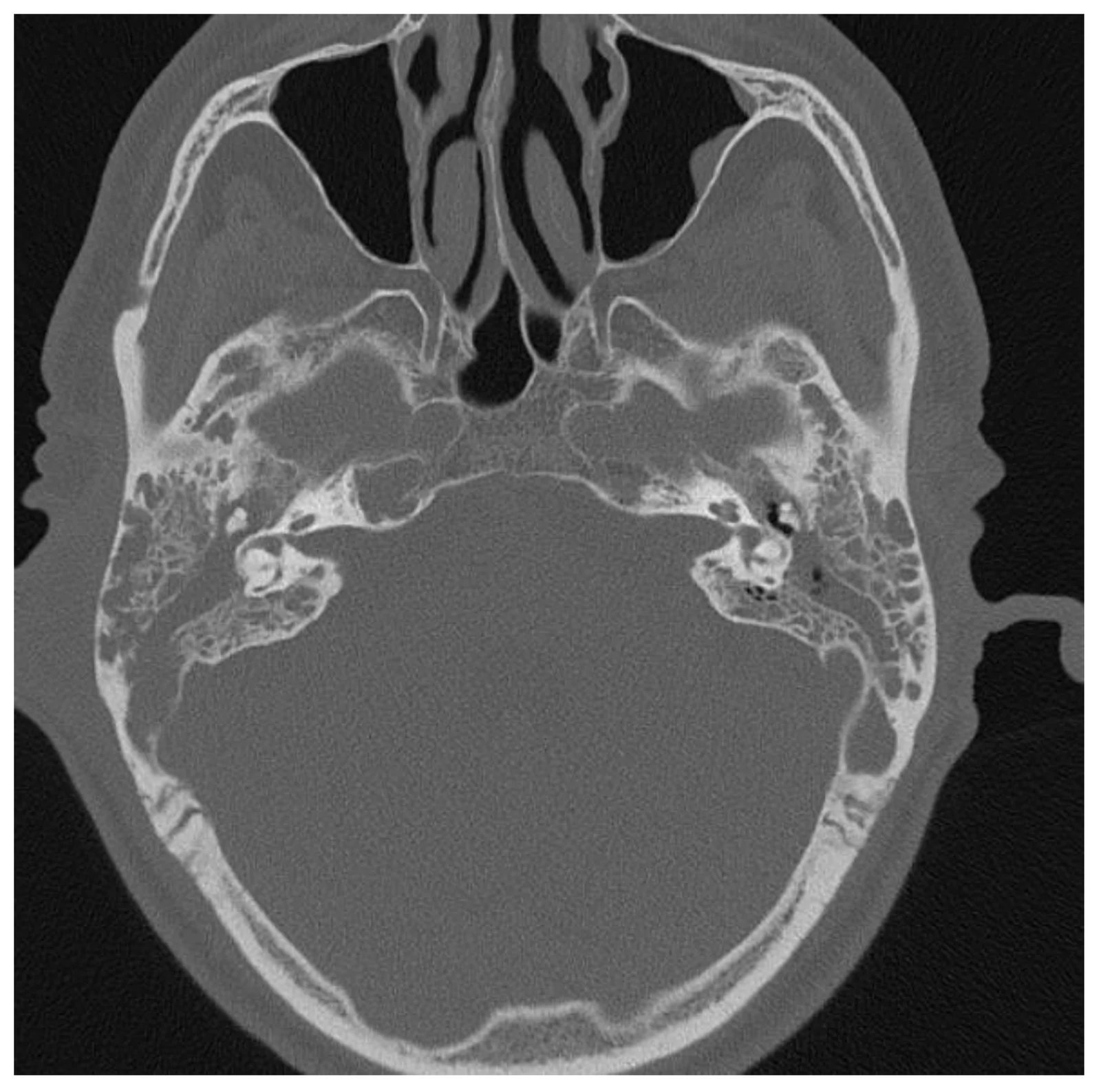
Axial CT of the temporal bones. The right side demonstrates complete opacification of the tympanic cavity and mastoid air cells, indicative of coalescent mastoiditis. There is significant destruction of the mastoid trabeculae and focal cortical erosion, specifically involving the sigmoid sinus plate. Marked postauricular soft tissue swelling is observed.

**Figure 2:**
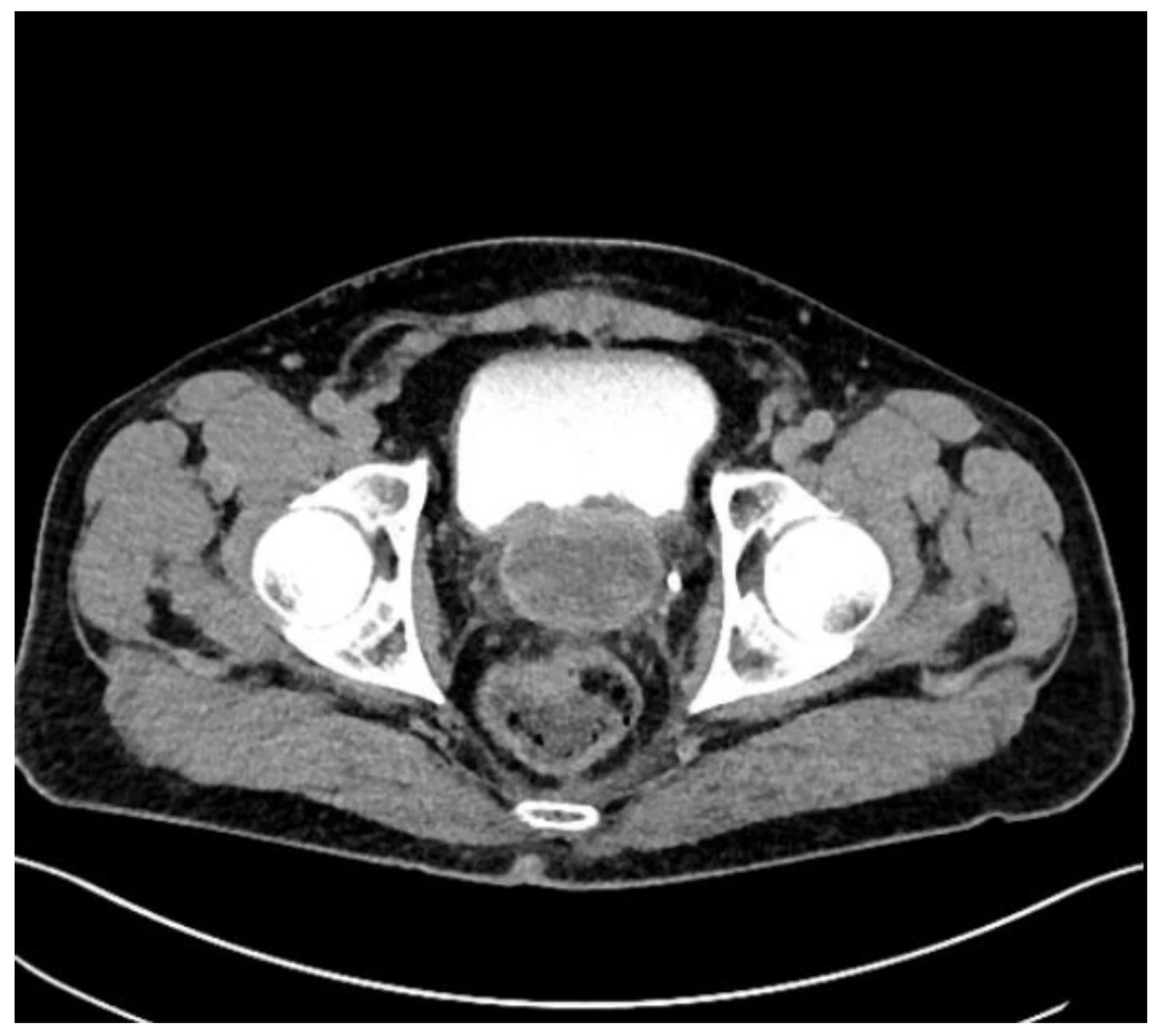
Axial contrast-enhanced CT of the pelvis. A well-circumscribed, irregular fluid collection is noted within the prostatic fossa, measuring 5.8 cm × 5.2 cm × 6.5 cm. The collection exhibits peripheral rim enhancement, characteristic of a prostatic abscess. The abscess appears to communicate with the urethra. Notably, there is irregular thickening of the posterior bladder wall adjacent to the collection.

**Figure 3:**
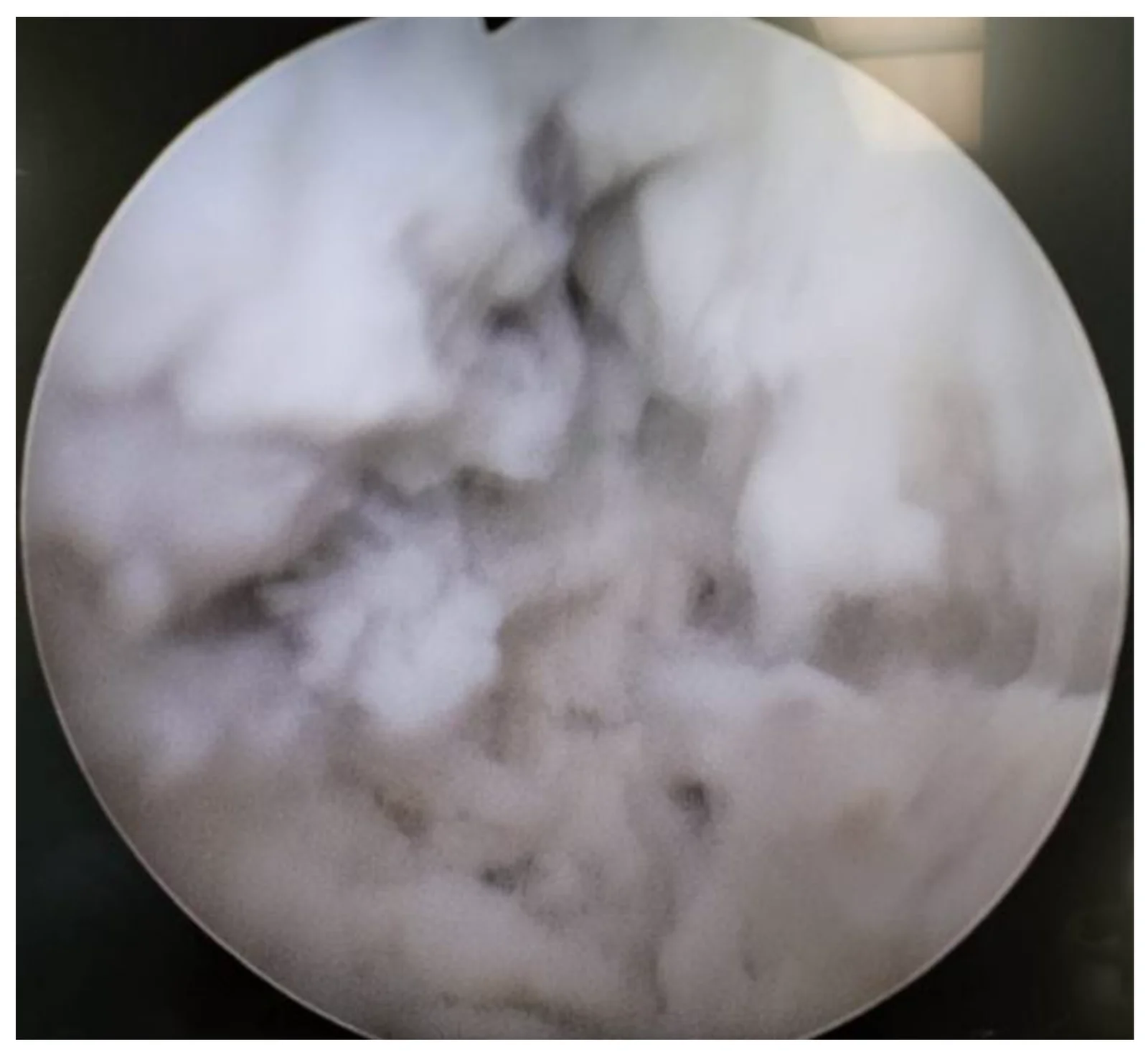
Endoscopic view of the prostatic urethra (proximal to the external sphincter). Massive fibrin deposits and necrotic debris are seen obliterating the prostatic segment, which precluded clear identification of the urethral lumen during the attempted transurethral drainage.

**Figure 4:**
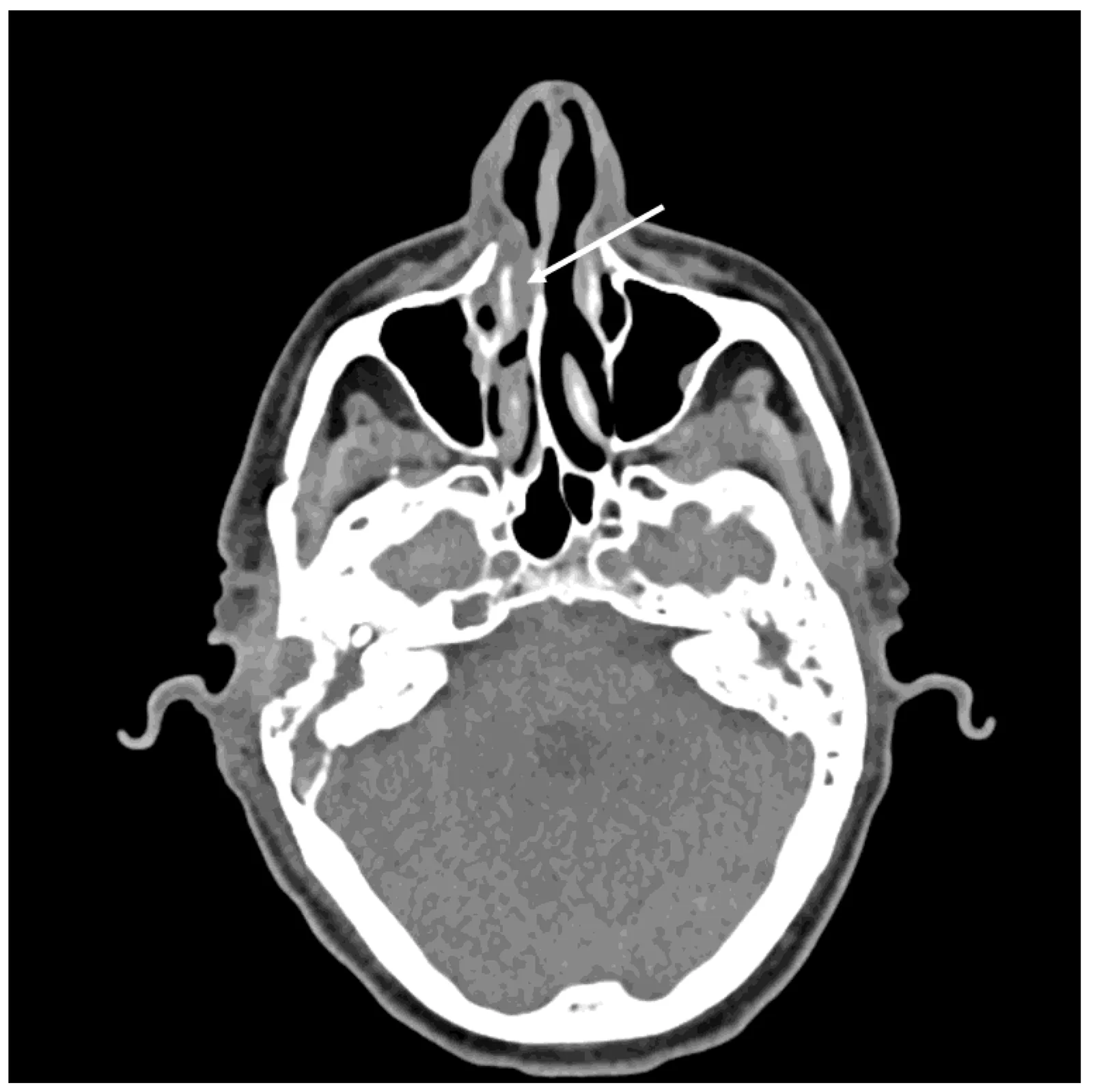
Axial contrast-enhanced maxillofacial CT. Nodular mucosal thickening of the nasal cavity (white arrow) and the right maxillary sinus ostium, both showing intense post-contrast enhancement.

**Figure 5:**
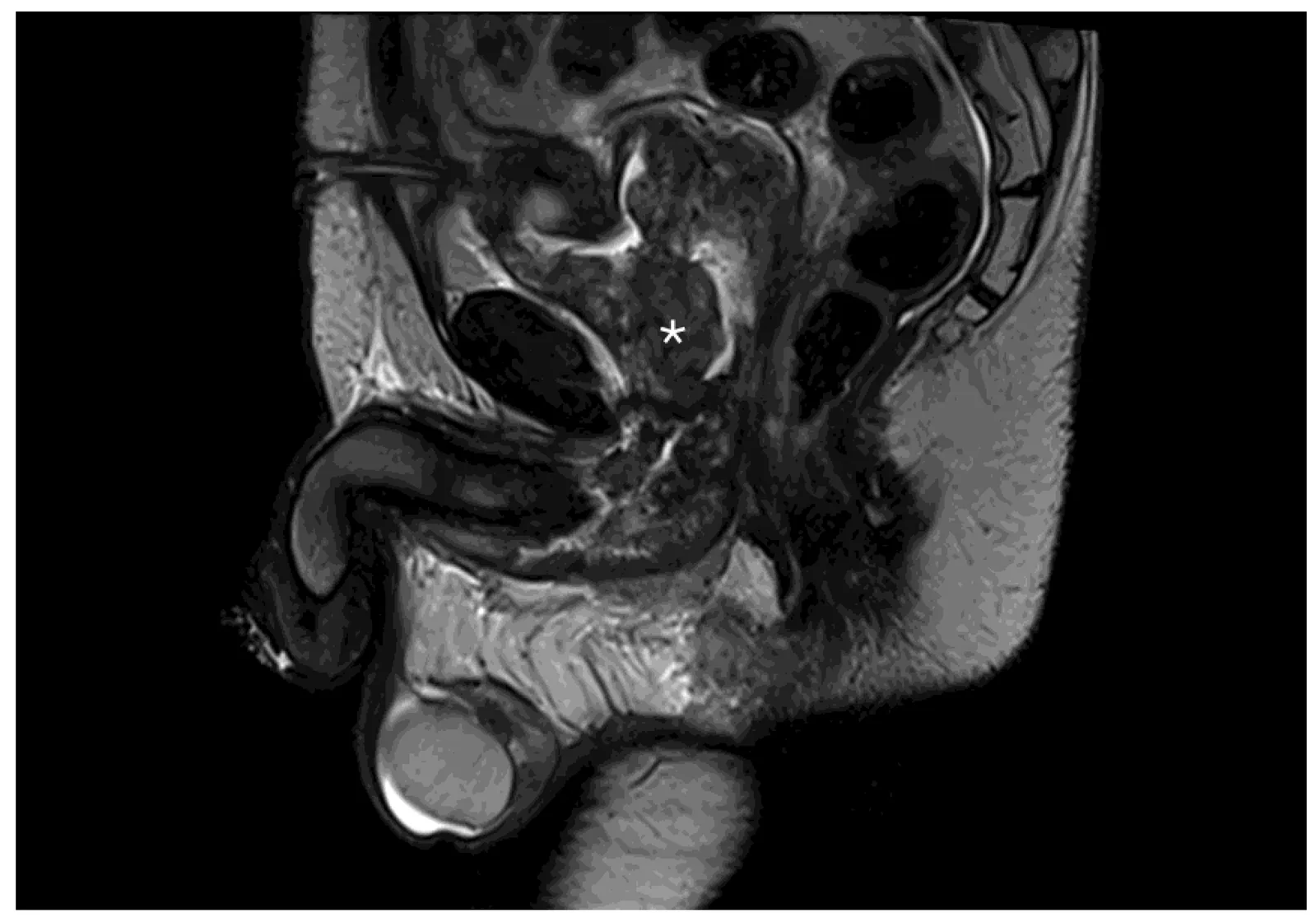
Sagittal T2-weighted MRI of the pelvis. An extensive, heterogeneously signal-intense inflammatory-necrotic collection (maximal dimensions 12 cm × 6.5 cm × 5.5 cm) is demonstrated. The lesion originates from the prostatic fossa (asterisk) and extends anteriorly and inferiorly to involve the bladder trigone, the penile bulb, and the base of the penis.

**Figure 6:**
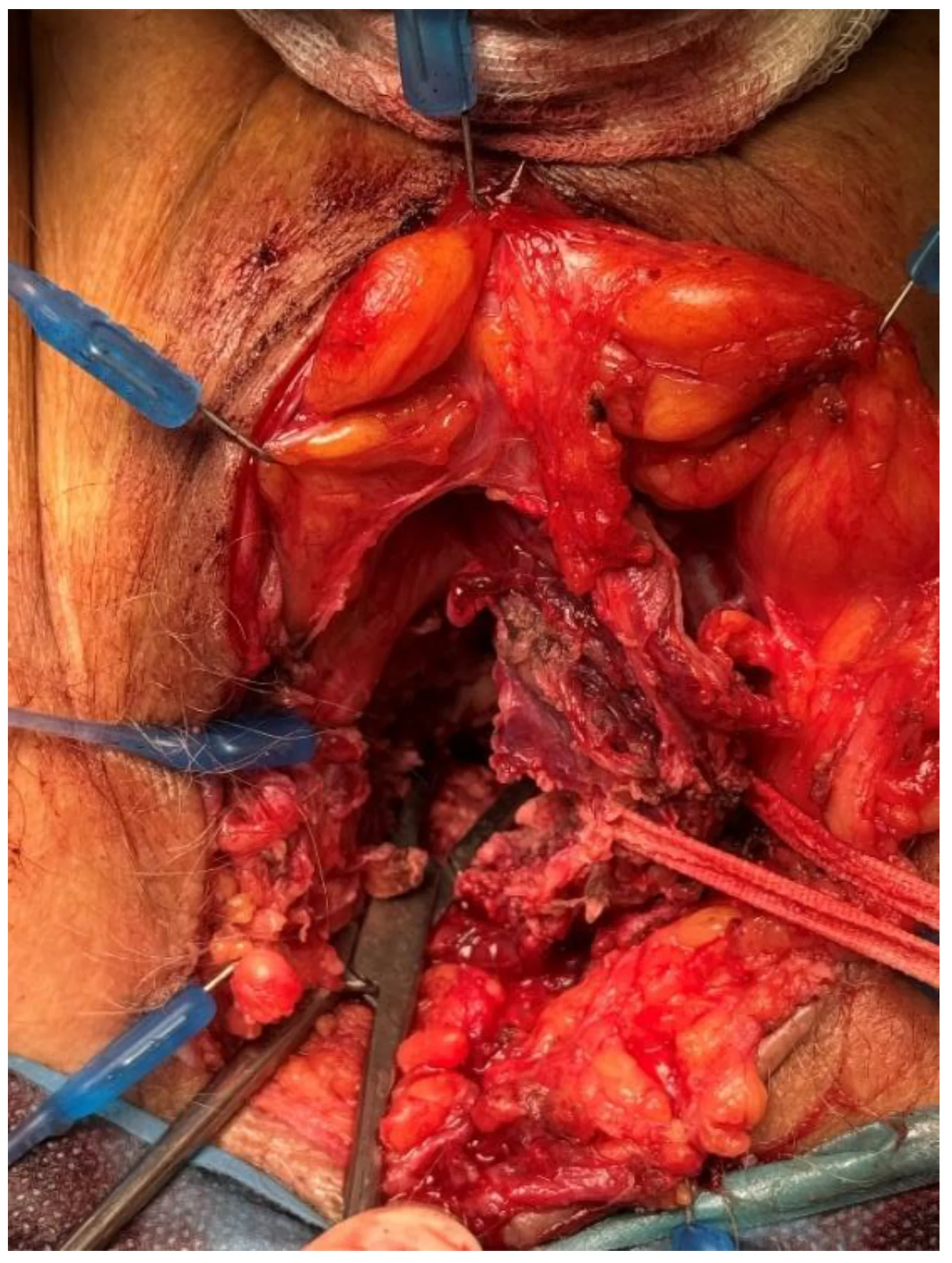
Open perineal drainage of the massive pelvic and prostatic abscess. Following the failure of the transurethral attempt, a deep, destructive inflammatory cavity is visualized. Abundant dark, necrotic debris and amorphous material are observed infiltrating the anatomical space corresponding to the urethral and prostatic fossa region.

**Figure 7:**
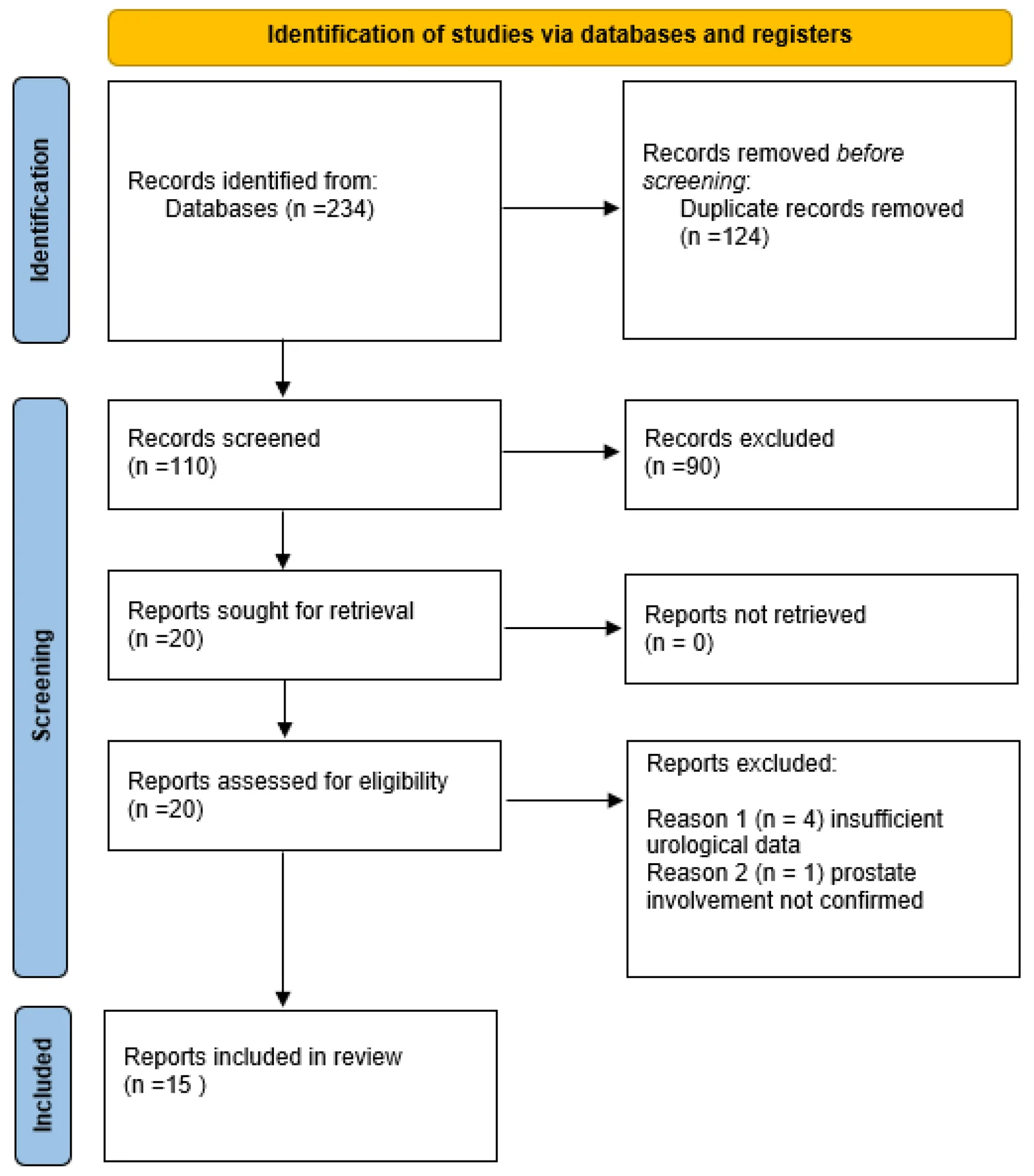
PRISMA-ScR flow diagram illustrating the study selection process.

**Table 1:** Clinical timeline of the present case (CARE-compliant). ANCA, antineutrophil cytoplasmic antibodies; AUR, acute urinary retention; BVAS/WG, Birmingham Vasculitis Activity Score for Wegener’s Granulomatosis; CRP, C-reactive protein; CYC, cyclophosphamide; ESBL, extended-spectrum β-lactamase; PCN, percutaneous nephrostomy; PCNL, percutaneous nephrolithotripsy; RTX, rituximab; TURP, transurethral resection of the prostate; UPJ, ureteropelvic junction; URS, ureterorenoscopy; UTI, urinary tract infection; VCUG, voiding cystourethrography.

Date	Event	Domain
November 2024	URS (external facility) for left hydronephrosis and suspected calculus; necrotic tissue in prostatic urethra; postoperative AUR → suprapubic cystostomy	Urological onset
December 2024	Onset of right-sided hearing loss and painful right postauricular swelling	Otological onset
January 2025	TURP for suspected prostatic abscess (external facility); histology: granulomatous inflammation with karyorrhectic necrosis; levofloxacin + metronidazole without improvement	Urology
February 2025	Admission, Dept. of Otolaryngology, UCC Gdańsk; CRP 96.3 mg/L, eosinophils 1.14 × 10^9^/L; CT: right subperiosteal mastoid abscess; right antromastoidectomy; cultures negative; histology: karyorrhectic necrosis, eosinophil-rich infiltrate	ENT
February–March 2025	Prostatic-fossa abscess 5.8 cm × 5.2 cm × 6.5 cm on CT; urine cultures, atypical-pathogen and TB testing negative; attempted transurethral drainage: lumen obliterated by necrosis → suprapubic cystostomy reinserted; histology: necrotic-karyorrhectic material, no malignancy	Urology
March 2025	Recurrent postauricular abscess: incision and drainage; CRP 183 mg/L; HRCT: progressive mastoid destruction; CT: sinonasal mucosal thickening; chest CT: calcified nodes unchanged since 2011; pelvic MRI: 12 cm × 6.5 cm × 5.5 cm collection from prostate to penile bulb/base and trigone, left UPJ infiltration with hydronephrosis; mini-PCN with nephroscopic biopsy of UPJ lesion (suppurative-necrotic inflammation, no malignancy)	ENT/Urology
April 2025	Open perineal drainage with resection of necrotic urethral and prostatic tissue; infectious, neoplastic and IgG4-related disease work-up negative; ANCA testing performed; prednisone started	Diagnosis
May 2025	Systemic ANCA-positive GPA diagnosed; first CYC pulse (1000 mg; 12.5 mg/kg, reduced for leukopenia)	Diagnosis/Treatment
June 2025	AUR two weeks after catheter removal → suprapubic cystostomy re-established; left orchitis (levofloxacin); CYC pulses	Urology/Treatment
July–September 2025	CYC pulses; VCUG: post-abscess cavity with polypoid mass, bulbar urethral stricture; ANCA testing; left nephrostomy for obstructing UPJ calculus	Treatment/Urology
October–November 2025	ESBL *P. aeruginosa* UTI → inpatient i.v. antibiotics; left PCNL with ureteral stent; retroauricular fistula closed surgically; RTX 1000 mg	Urology/ENT/Treatment
December 2025	RTX 1000 mg	Treatment
March–April 2026	Pelvic MRI (Mar) and CT (Apr): complete regression of prostatic-fossa collection, no residual abscess	Imaging follow-up
May–June 2026	RTX 1000 mg; clinical remission (BVAS/WG 0); suprapubic cystostomy in situ	Follow-up

**Table 2:** Summary of post-2019 reported cases of prostatic involvement in GPA and the present case (*n* = 16). Abbreviations: PI, prostatic involvement; AKI, acute kidney injury, AUR, acute urinary retention; LUTS, lower urinary tract symptoms; BOO, bladder outlet obstruction; UTI, urinary tract infection; ENT, ear, nose, and throat; CNS, central nervous system; PNS, peripheral nervous system; ANCA, antineutrophil cytoplasmic antibody; c-ANCA, cytoplasmic ANCA; p-ANCA, perinuclear ANCA; PR3, proteinase 3; MPO, myeloperoxidase; FDG, fluorodeoxyglucose; PET, positron emission tomography; GC, glucocorticoids; RTX, rituximab; CYC, cyclophosphamide; AZA, azathioprine; MTX, methotrexate; MMF, mycophenolate mofetil; TURP, transurethral resection of the prostate; PCN, percutaneous nephrostomy; UPJ, ureteropelvic junction; CR, complete remission; N/A, not available; Symbols: (+), positive result; (−), negative result.

Author	Year of Publication	Age of PI	Time to GPA Diagnosis	Presenting Symptoms Related to PI	PI: Urological Symptoms	PI: Imaging Findings	Other Organ Involvement	ANCA Profile	Immunosuppressive Treatment	Surgical Treatment (Prostate-related)	Outcome
Chevalier [14]	2020	55	1/2 month	Yes	Dysuria, AUR, pollakiuria	Prostatic abscess, prostatic oedema	Lungs, joints, eyes, parotid gland	c-ANCA (+)/p-ANCA N/A/PR3 (+)/MPO N/A	GC, RTX	Transrectal abscess drainage	Improvement at 10 days; no follow-up
Neerhut [15]	2021	78	9 months	Yes	Progressive LUTS	Prostatic enlargement, focal intraprostatic lesion	Lungs, ENT	c-ANCA (+)/p-ANCA N/A/PR3 N/A/MPO N/A	GC, RTX	TURP ×2	Died—cause uncertain
García-Guevara [16]	2021	58	Not applicable- prostatic involvement identified during relapse	No	Dysuria, haematuria, pollakiuria, urinary incontinence	Prostatic calcifications	Lungs, ENT, eyes, CNS, PNS, systemic	c-ANCA (+)/p-ANCA N/A/PR3 (+)/MPO N/A	GC, CYC, AZA	None	Improvement at 6 weeks
Trives-Folguera [17]	2022	66	Not reported	Yes	BOO, haematuria, urethral bleeding (relapse)	Not performed	Skin	c-ANCA (+)/p-ANCA N/A/PR3 (+)/MPO N/A	GC, MTX, CYC, AZA, RTX (across initial + 2 relapses)	None	Improvement; ongoing steroid taper
Ponnuru [18]	2022	58	<1 month	Yes	AUR, pyuria	Prostatic abscesses, prostatic enlargement	Lungs, ENT, joints, systemic, AKI without histological confirmation	c-ANCA (+)/p-ANCA N/A/PR3 (+)/MPO N/A	GC, CYC, plasmapheresis	TURP	Died—diffuse alveolar haemorrhage
Clerc [19]	2022	46	3 months	Yes	Dysuria, AUR, haematuria, recurrent UTI	Prostatic abscess, intense FDG uptake on PET	Lungs, ENT, systemic, bladder	c-ANCA (+)/p-ANCA N/A/PR3 (+)/MPO N/A	GC, CYC, AZA	None	Remission at 24 months; radiological CR
Butt [20]	2022	44	8 months	Yes	Dysuria, intermittent urinary retention, haematuria, pyuria, pelvic pain	Not performed	Lungs, ENT, skin, systemic	c-ANCA (+)/p-ANCA (−)/PR3 N/A/MPO N/A	GC, RTX, MTX	TURP	Improvement; follow-up duration not reported
Kimbrough [21]	2023	63	3 months	Yes	Chronic urinary retention	Prostatic enlargement	Eyes, systemic, muscles	c-ANCA N/A/p-ANCA (+)/PR3 N/A/MPO (+)	GC, RTX	None	Improvement, no further follow-up
Jain [22]	2024	26	1/2 month	No	Asymptomatic	Prostate lesions	Lungs, spleen, systemic	c-ANCA N/A/p-ANCA N/A/PR3 (+)/MPO (−)	GC, RTX, AZA	None	Remission at 12 months
Rashid [23]	2024	36	1 month	Yes	Dysuria, AUR, LUTS, urethral discharge	Prostatic abscess, urethral necrosis, ano-urethral fistula	Lungs	c-ANCA (+)/p-ANCA N/A/PR3 (+)/MPO N/A	GC, RTX, MMF	TURP, incision and drainage of perianal abscess, suprapubic catheter insertion	Improvement; fistula healed on imaging
Ince [24]	2024	46	Not reported	No	Dysuria, haematuria, pyuria	Prostatic abscess	Lungs, ENT, kidneys, CNS, systemic	c-ANCA N/A/p-ANCA N/A/PR3 (+)/MPO N/A	GC, CYC, RTX	Drainage of prostate abscess fluid	Remission at 5 years; CNS relapse resolved
Barutcu Atas [25]	2025	60	3 months	No	Asymptomatic	Not performed	Lungs, kidneys, systemic	c-ANCA (+)/p-ANCA N/A/PR3 (+)/MPO N/A	GC, RTX	None	Improvement; follow-up duration not reported
Kamada [26]	2025	71	6 months	Yes	Haematuria, pollakiuria, nocturia, BOO	Prostatic abscess, bladder fistula	Lungs, ENT, kidneys, CNS, eyes, systemic, spleen	c-ANCA N/A/p-ANCA N/A/PR3 (+)/MPO (−)	GC	Urinary diversion	Died—intracranial haemorrhage (day 13)
Kontorshchikov [27]	2025	62	2 months	Yes	Dysuria, pollakiuria, BOO	Prostatic abscess, prostate nodules, prostatic enlargement	Lungs, kidneys, bladder, systemic	c-ANCA N/A/p-ANCA N/A/PR3 (+)/MPO (−)	GC	Abscess drainage, cystostomy	Died—cardiac arrest (one day after GPA diagnosis)
Zhang [28]	2026	53	9 months	Yes	Pollakiuria, urgency	Prostatic enlargement, widening of the urethra with multiple lesions	Lungs, ENT	c-ANCA (+)/p-ANCA N/A/PR3 (+)/MPO N/A	GC, CYC	None	Remission at 6 months
Present case	2026	60	7 months	Yes	Dysuria, AUR, pelvic pain, urethral discharge	Prostatic abscess, urethral necrosis, bladder trigone and penile involvement, left UPJ infiltration	ENT, systemic	c-ANCA (+)/p-ANCA (−)/PR3 (+)/MPO (−)	GC, CYC, RTX	TURP, suprapubic cystostomy, PCN, open perineal drainage with urethral resection	Clinical remission (BVAS/WG 0 at 13 months); persistent urethral stricture, suprapubic diversion, recurrent urinary retention, UTIs, left nephrostomy, left orchitis, Th7 fracture

## Data Availability

The data presented in this study are available on request from the corresponding author due to privacy and ethical restrictions applying to individual patient clinical data.

## Supplementary Materials

The following supporting information can be downloaded at: https://www.mdpi.com/article/10.3390/diagnostics16193101/s1. Supplementary Material S1: Full electronic search strategies; Table S1: PRISMA-ScR checklist [13].
