# Supplementary material for: Prostatic Involvement in Granulomatosis with Polyangiitis: A Case Report and Scoping Review
Source: Diagnostics (Basel). 2026 Sep 24;16(19):3101. doi: 10.3390/diagnostics16193101 (PMC13664353; doi:10.3390/diagnostics16193101)
Supplement: Supplementary file 1 [file diagnostics-16-03101-s001.zip › Supplementary_Material_S1.pdf]

Supplementary Material S1. Full electronic search strategies

Manuscript: Prostatic Involvement in Granulomatosis with Polyangiitis: A Case Report and Scoping Review.

In accordance with PRISMA-ScR item 8, the full electronic search strategy is presented for every database exactly as executed, with all limits and the number of records retrieved, so that each search can be reproduced. Strategies 1 and 2 were executed for the original submission; strategies 1b, 2b, 3 and 4 were added during peer review.

Database 1 — PubMed (searched 30 April 2026)

```
("granulomatosis with polyangiitis"[Title/Abstract] OR "Wegener's
granulomatosis"[Title/Abstract] OR "ANCA-associated vasculitis"[Title/Abstract]) AND
(prostate[Title/Abstract] OR prostatic[Title/Abstract] OR prostatitis[Title/Abstract])
```

Limits: search fields restricted to Title/Abstract; no language restriction; publication date 2019–2026 (publication-date filter). Records retrieved: 22.

Database 1b — PubMed, re-executed with apostrophe variants and controlled vocabulary (searched 24 August 2026)

```
("Granulomatosis with Polyangiitis"[Mesh] OR "granulomatosis with
polyangiitis"[Title/Abstract] OR "Wegener's granulomatosis"[Title/Abstract] OR "Wegener
granulomatosis"[Title/Abstract] OR "Wegeners granulomatosis"[Title/Abstract] OR "Anti-
Neutrophil Cytoplasmic Antibody-Associated Vasculitis"[Mesh] OR "ANCA-associated
vasculitis"[Title/Abstract] OR "ANCA associated vasculitis"[Title/Abstract]) AND
("Prostate"[Mesh] OR "Prostatic Diseases"[Mesh] OR "Prostatitis"[Mesh] OR
prostate[Title/Abstract] OR prostatic[Title/Abstract] OR prostatitis[Title/Abstract]) AND
("2019/01/01"[Date - Publication] : "3000"[Date - Publication])
```

Limits: as above. Records retrieved: 22; records not retrieved by strategy 1: 0.

Database 2 — Scopus (searched 30 April 2026)

```
( TITLE-ABS-KEY ( "granulomatosis with polyangiitis" OR "Wegener granulomatosis" OR
"ANCA-associated vasculitis" ) ) AND ( TITLE-ABS-KEY ( prostate OR prostatic OR
prostatitis ) ) AND PUBYEAR > 2018 AND PUBYEAR < 2027
```

Limits: TITLE-ABS-KEY (title, abstract, keywords); publication years 2019–2026; no language restriction. Records retrieved: 91.

Database 2b — Scopus, re-executed with apostrophe variants (searched 24 August 2026)

```
( TITLE-ABS-KEY ( "granulomatosis with polyangiitis" OR "Wegener granulomatosis" OR
"Wegener's granulomatosis" OR "Wegeners granulomatosis" OR "ANCA-associated vasculitis"
OR "ANCA associated vasculitis" ) ) AND ( TITLE-ABS-KEY ( prostate OR prostatic OR
prostatitis ) ) AND PUBYEAR > 2018 AND PUBYEAR < 2027
```

Limits: as for Database 2. Records retrieved: 91; records not retrieved by strategy 2: 0.

Database 3 — Web of Science Core Collection (searched 24 August 2026)

```
TS=("granulomatosis with polyangiitis" OR "Wegener granulomatosis" OR "Wegener's
granulomatosis" OR "Wegeners granulomatosis" OR "ANCA-associated vasculitis" OR "ANCA
associated vasculitis") AND TS=(prostate OR prostatic OR prostatitis)
```

Refined by: Publication Years = 2019 OR 2020 OR 2021 OR 2022 OR 2023 OR 2024 OR 2025 OR 2026.

Limits: Topic field (title, abstract, author keywords, Keywords Plus); editions: SCI-EXPANDED, SSCI, A&HCI, ESCI, CPCI-S, CPCI-SSH, BKCI-S, BKCI-SSH, CCR-EXPANDED, IC; publication years 2019–2026; no language restriction. Records retrieved: 25.

Database 4 — Embase (Elsevier, Embase.com; searched 24 August 2026)

```
('wegener granulomatosis'/exp OR 'granulomatosis with polyangiitis':ti,ab,kw OR 'wegener
granulomatosis':ti,ab,kw OR 'wegeners granulomatosis':ti,ab,kw OR 'anca associated
vasculitis'/exp OR 'anca-associated vasculitis':ti,ab,kw OR 'anca associated
vasculitis':ti,ab,kw) AND ('prostate'/exp OR 'prostate disease'/exp OR 'prostatitis'/exp
OR prostate:ti,ab,kw OR prostatic:ti,ab,kw OR prostatitis:ti,ab,kw) AND [2019-2026]/py
NOT [conference abstract]/lim
```

Limits: title/abstract/keyword fields plus Emtree headings (exploded); publication years 2019–2026; no language restriction; conference abstracts excluded at the search stage ([conference abstract]/lim). Records retrieved: 96.

Record accounting

Combined records before deduplication: 234 (PubMed 22, Scopus 91, Web of Science 25, Embase 96; the re-executed PubMed and Scopus strategies 1b and 2b retrieved no records beyond those of strategies 1 and 2). Duplicates removed: 124. Records screened by title and abstract: 110. Records excluded at screening: 90. Reports assessed in full text: 20. Reports excluded at full text: 5 (insufficient urological data, n = 4; prostatic involvement not confirmed, n = 1). Reports included: 15. See Figure 7 (main text) for the PRISMA-ScR flow diagram.

Compared with the original submission (PubMed and Scopus only: 113 records, 21 duplicates, 92 screened, 19 assessed in full text, 4 excluded, 15 included), the extended search identified one additional report for full-text assessment, which was excluded for insufficient urological data; the set of included reports is unchanged.

Deduplication was performed in Zotero by automated matching of DOI, title and first author, followed by manual verification of all flagged pairs.

All 15 included reports were published in 2020 or later; none had been included in the review by Yatsyshyn et al. (2019).
